# Supplementary material for: The genome sequence of the Portuguese Dappled White, Euchloe tagis (Hübner, 1804) (Lepidoptera: Pieridae)
Source: Wellcome Open Res. 2026 Feb 9;11:100. [Version 1] doi: 10.12688/wellcomeopenres.25883.1 (PMC13100615; doi:10.12688/wellcomeopenres.25883.1)
Supplement: Peer review history

# Reviewer report: Reviewer response for version 1

**DOI:** 10.21956/wellcomeopenres.28490.r152546

**Published:** 2026-04-23

**Authors:** Tianzhu Xiong

**Affiliations:** 1 Cornell University, Ithaca, New York, USA

This report of the Euchloe tagis genome covers an important species of conservation value. A great amount of natural history is introduced, followed by a detailed method section on genome assembly strategies. The final assembly is at the chromosome level.

 My only question is why only one haplotype is selected to be a chromosome-level assembly? It seems that hap2 has a smaller number of contigs and scaffolds, with similar N50. The total length difference between hap1 and hap2 is only 1Mb. Is there any intrinsic reason that hap2 is not called a chromosome-level assembly?

 Apart from this minor point, the report is pretty complete and should be good to index.

Are sufficient details of methods and materials provided to allow replication by others?

Yes

Is the rationale for creating the dataset(s) clearly described?

Yes

Are the datasets clearly presented in a useable and accessible format?

Yes

Are the protocols appropriate and is the work technically sound?

Yes

Reviewer Expertise:

Genetics Genomics

I confirm that I have read this submission and believe that I have an appropriate level of expertise to confirm that it is of an acceptable scientific standard.

# Reviewer report: Reviewer response for version 1

**DOI:** 10.21956/wellcomeopenres.28490.r152543

**Published:** 2026-04-21

**Authors:** Gurinder Kaur Walia

**Affiliations:** 1 Punjabi University, Patiala, Punjab, India

This manuscript presents a high-quality chromosome-level genome assembly of *Euchloetagis*. The study is well-designed, and the methodologies used are robust and appropriate. The assembly metrics are excellent, with high completeness and strong chromosomal resolution, making this a valuable genomic resource for Lepidoptera research.

 The manuscript is clearly written, and the data are well presented and accessible. The integration of Hi-C scaffolding and Merian element mapping further strengthens the study.

**Minor suggestions:**

- The manuscript primarily focuses on how the genome was sequenced and the quality of the assembly.
- However, relatively limited emphasis is given to the biological insights derived from the genome.
- A brief addition on how this genome facilitates comparative analysis of *Euchloe* with other butterfly taxa would enhance the study.
- Inclusion of insights into evolutionary history, adaptive mechanismsand host-plant associations would further strengthen the manuscript.
- Highlighting its relevance for ecological studies and conservation applications would improve the overall impact.
- Minor typographical inconsistencies should be corrected, particularly the spelling of “Portuguese,” which appears as both “Portuguese” and “Portugese” in the manuscript.

**Recommendation:**

- Accept with minor revisions.

Are sufficient details of methods and materials provided to allow replication by others?

Yes

Is the rationale for creating the dataset(s) clearly described?

Yes

Are the datasets clearly presented in a useable and accessible format?

Yes

Are the protocols appropriate and is the work technically sound?

Yes

Reviewer Expertise:

molecular cytogenetics

I confirm that I have read this submission and believe that I have an appropriate level of expertise to confirm that it is of an acceptable scientific standard.

# Reviewer report: Reviewer response for version 1

**DOI:** 10.21956/wellcomeopenres.28490.r152144

**Published:** 2026-04-21

**Authors:** Temitope Opeyemi Oriowo

**Affiliations:** 1 Leibniz Institute for the Analysis of Biodiveristy Change, Germany, Germany

- Were other K-mers tested? I always recommend trying some different kmers and then deciding which has the lowest error rate

 - Overall, I think it is a well-written genome note, and aside from being a nice resource to have, especially in the face of such taxonomic uncertainty, it is technically sound too.

 - All linked resources are available too

 -Article approved.

Are sufficient details of methods and materials provided to allow replication by others?

Yes

Is the rationale for creating the dataset(s) clearly described?

Yes

Are the datasets clearly presented in a useable and accessible format?

Yes

Are the protocols appropriate and is the work technically sound?

Yes

Reviewer Expertise:

I work in population genomics, and genome assemblies are part of what I do

I confirm that I have read this submission and believe that I have an appropriate level of expertise to confirm that it is of an acceptable scientific standard.

# Reviewer report: Reviewer response for version 1

**DOI:** 10.21956/wellcomeopenres.28490.r148587

**Published:** 2026-03-12

**Authors:** Melody Clark

**Affiliations:** 1 Natural Environment Research Council, Cambridge, UK

This Data Note describes the genome sequencing of the Portuguese Dappled White, *Euchloe tagis *under the Project Psyche. The biology and biogeography of this butterfly is well described, as is the rationale for genome sequencing. Although this butterfly is listed as "Least Concern" under the IUCN Red List, it is threatened by habitat loss and overgrazing in some European regions. It is also highly constrained by its specificity to its host plants and the geological characteristics of the preferred area, namely rocky limestone. The genome sequence of a male specimen is of very high quality, comprising 30 pseudochromosomes and the Z sex chromosome, plus the mitochondrial genome. The DNA extraction and sequencing methodologies, plus assembly and annotation are well described and use standard protocols and tools that are in the public domain. The genome has been annotated by Ensembl. All data are in the public domain. There is also mapping data of Merian elements, relating this genome to the 32 ancestral linkage groups of Lepidoptera.

Are sufficient details of methods and materials provided to allow replication by others?

Yes

Is the rationale for creating the dataset(s) clearly described?

Yes

Are the datasets clearly presented in a useable and accessible format?

Yes

Are the protocols appropriate and is the work technically sound?

Yes

Reviewer Expertise:

transcriptomics and genomics of non-model species

I confirm that I have read this submission and believe that I have an appropriate level of expertise to confirm that it is of an acceptable scientific standard.
